# Monosodium Glutamate (MSG) Renders Alkalinizing Properties and Its Urinary Metabolic Markers of MSG Consumption in Rats

**DOI:** 10.3390/biom9100542

**Published:** 2019-09-27

**Authors:** Kanokwan Nahok, Jia V. Li, Jutarop Phetcharaburanin, Hasina Abdul, Chaisiri Wongkham, Raynoo Thanan, Atit Silsirivanit, Sirirat Anutrakulchai, Carlo Selmi, Ubon Cha’on

**Affiliations:** 1 Department of Biochemistry, Faculty of Medicine, Khon Kaen University, Khon Kaen 40002, Thailand; Kanokwan.nahok@gmail.com (K.N.); jutarop@kku.ac.th (J.P.); chaisiri@kku.ac.th (C.W.); raynoo@kku.ac.th (R.T.); atitsil@kku.ac.th (A.S.); 2 Chronic Kidney Disease prevention in the Northeast Thailand (CKDNET), Khon Kaen University, Khon Kaen 40002, Thailand; sirirt_a@kku.ac.th; 3 Department of Metabolism, Digestive Disease and Reproduction, Faculty of Medicine, Imperial College London, South Kensington, London SW7 2AZ, UK; jia.li@imperial.ac.uk (J.V.L.); hasinaabdul@hotmail.co.uk (H.A.); 4 Centre for Digestive and Gut Health, Institute of Global Health Innovation, Imperial College London, London SW7 2AZ, UK; 5 Department of Medicine, Faculty of Medicine, Khon Kaen University, Khon Kaen 40002, Thailand; 6 Rheumatology and Clinical Immunology, IRCCS Humanitas Clinical and Research Center, 20089 Milan, Italy; 7 BIOMETRA Department, University of Milan, 20089 Milan, Italy

**Keywords:** monosodium glutamate, metabolic profiles, alkaline urine, ion exchangers

## Abstract

Monosodium glutamate (MSG) is widely used as a flavor enhancer and its effects on human health are still debated. We aimed to investigate whether MSG can act as alkalinizing agent in murine models and if its metabolites are biomarkers of MSG consumption. For this purpose, adult male Wistar rats were given water added with 1 g% MSG or three types of control water, including sodium chloride (NaCl) and sodium bicarbonate (NaHCO_3_). At 14 days, urinary pH, electrolytes, urinary metabolites and ion-exchanger gene expression were determined. The results revealed that MSG-treated rats had significantly more alkaline urine and higher levels of urinary sodium and bicarbonate similar to NaHCO_3_ controls. These changes correlated with a lower expression of ion-exchanger genes, namely, *CAII, NBC1*, and *AE1*, which are involved in bicarbonate kidney reabsorption. The urinary metabolic profiles also revealed similar patterns for the MSG and NaHCO_3_ groups. In conclusion, MSG exhibits similar properties to NaHCO_3_, an alkalinizing agent, with regard to inducing alkaline urine, reducing bicarbonate kidney reabsorption, and generating a specific urinary metabolic pattern. We believe that these observations will be useful to further study the MSG effects in humans.

## 1. Introduction

Monosodium glutamate (MSG) is commercially produced as a flavor enhancer in processed foods and home cooking and its use is exponentially increasing worldwide [1]. Although the Food and Drug Administration (FDA) have classified MSG as a safe food ingredient [2], its safety as a food additive is still debated. For example, cross-sectional and longitudinal studies from our group and others have revealed that the consumption of MSG is associated with human metabolic syndrome [3], obesity [4,5], and arterial hypertension [6], although conflicting evidence is also available [7,8].

The metabolic effects of both oral and parenteral MSG seem to be consistent in animal models, with data available on the effects of MSG on the liver [9,10,11], pancreas [12,13] and kidney [9,14]. MSG causes alkaline urine in rats following long-term consumption [14]. Urine alkalinization using available alkalinizing agents such as potassium citrate (K citrate) [15,16] and sodium bicarbonate (NaHCO_3_) [17,18] is a common treatment for several medical conditions, with the latter used for treating patients who have acute metabolic acidosis or renal proximal tubular acidosis [19]. Of importance, potassium citrate has limitations in patients with hyperkalemia [20,21,22] and in long-term supplementation, and NaHCO_3_ may lead to adverse events such as systemic alkalosis [19].

Since alkalinizing agents should be used with caution in specific subjects, it is very important to identify whether MSG is also able to alkalinize urine, even though our previous observations revealed that a 9-month exposure to MSG did not affect the blood pH [14]. We herein report that oral MSG (resembling human intake) can alkalinize urine within two weeks and our ^1^H-NMR-based metabolomics approach revealed that MSG induced urinary metabolic profiles were similar to those of NaHCO_3_, an alkalinizing agent. We ultimately submit that urinary metabolites may be useful for monitoring dietary consumption, and thus predict MSG effects in human.

## 2. Materials and Methods

### 2.1. Chemicals and Animals

We used 99% pure food-grade MSG (Ajinomoto, Japan), NaCl (RCI Labscan, Thailand), NaHCO_3_ (BDH, Visalia, CA, USA) and 40 6-week old male Wistar rats (approximately 200 g) obtained from the National Laboratory Animal Center (Salaya, Mahidol University, Bangkok, Thailand). Animals were housed for 2 weeks in the Northeast Laboratory Animal Center and maintained under standard conditions (temperature: 23 ± 2 °C, humidity (RH): 30–60%, brightness: 350–400 Lux and 12 h/12 h dark/light cycle) with standard rat chow pellets (Perfect Companion Group, Bangkok, Thailand). All experiments were performed in accordance with the guidelines of KKU animal ethics (AEKKU-NELAC 5/2558).

### 2.2. Experimental Design

**Figure 1:**
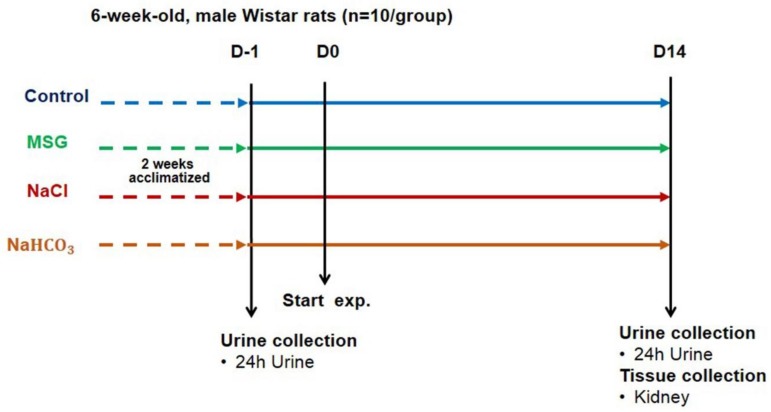
A schematic diagram showing the experimental design and sample collection.

Rats were assigned into 4 groups, (1) receiving drinking water, (2) receiving water with 1 g% MSG added, (3) receiving water with 0.34 g% NaCl added, which equivalent to the sodium in group 2, and (4) receiving water with 2.4 g% NaHCO_3_ as a positive control for the alkalinizing agent treated group (Figure 1). Animals were allowed to access water and food ad libitum and daily water intake was recorded weekly. Food intake (g/day) was measured every 2 weeks. Rats were housed in individual stainless steel metabolic cages for urine sample collection. Urinary samples (24-h urine) were collected one day before (D1) and 14 days after the experiment (D14) and kept at −80 °C until analyzed. At the end of the experiment (D14), all animals were sacrificed using carbon dioxide after 12-h fasting. Kidneys were dissected, washed with normal saline, and divided into cortex and medulla layer, then transferred to cryotubes with TRIzol^®^ Reagent (Invitrogen, Waltham, MA, USA), dipped in liquid nitrogen and stored at −80 °C for gene expression analysis.

### 2.3. Urine Analysis

The 24 h urine was thawed at room temperature and analyzed for urine pH and urine electrolyte (sodium, potassium, chloride and bicarbonate). All samples were analyzed at the laboratory services in Srinagarind Hospital, Khon Kaen University and using automatic machine under standard protocol.

### 2.4. Sample Preparation for ^1^H-NMR Spectroscopic Analysis

Urine samples were thawed at room temperature and centrifuged at 18,000× *g*, 4 °C, for 10 min. Then 100 µL of the urine supernatant was transferred to a new tube and mixed with 300 µL of deuterium oxide (D_2_O) (Sigma Aldrich, Gillingham Dorset, UK) and 250 µL of 0.2 M sodium phosphate buffer containing 0.1% sodium trimethylsilyl-[2,2,3,3-^2^H_4_]-propionate (TSP), 100% D_2_O and 3 mM sodium azide. The sample was vortexed briefly, and then 600 µL of the mixture was transferred to NMR glass tubes (Wilmad-labglass, Vineland, NJ, USA) with an outer diameter of 5 mm pending NMR analysis.

### 2.5. ^1^H-Nuclear Magnetic Resonance Spectroscopic Analysis of Urine

Urinary samples were analyzed using a Bruker 600 MHz spectrometer (Bruker Avance III, Bruker Biospin, Rheinstetten, Germany) at a ^1^H frequency of 600.13 MHz with a temperature of 300 K. A standard one-dimensional (1-D) NMR pulse sequence (recycle delay [RD]-90°-t_1_-90°-t_m_-90°-acquire free induction decay) was used t_1_ was fixed to 3 µs. The suppression of the water peak was achieved by selective irradiation during recycle delay of 2 s and mixing time (t_m_) of 100 ms. The 90 degree-pulse was adjusted to approximately 10 µs. A total of 128 scans were recorded into 64 K data points with a spectral width of 20 ppm [23].

### 2.6. Data Pre-Processing and Multivariate Statistical Analysis

The urinary spectra were calibrated to the TSP peak (δ^1^H 0.00), phased and baseline corrected using TopSpin 3.0 (Bruker, Rheinstetten, Germany). The pre-processed spectra were imported into MATLAB software (MathWorks, R2014a, Natrick, MA, USA) and digitized with a resolution of 0.0005 ppm. Spectral regions containing the TSP peak (δ^1^H −1.00–0.005), water (δ^1^H 4.70–4.90) and urea (δ^1^H 5.71–6.00) were removed. Normalization using the probabilistic quotient method [24] and peak alignment using recursive segment-wise peak alignment (RSPA) [25] were applied to the remaining spectral data. Unsupervised principal component analysis (PCA) with unit variance (UV) scaling method was initially performed in order to identify any obvious clustering or outliers to unassigned treatment groups. The orthogonal-signal correction-projection to latent structures-discriminant analysis (O-PLS-DA) was then used to investigate the metabolic difference between the control and MSG groups. The O-PLS-DA models are evaluated by the R^2^X and Q^2^Y values, representing the fitness and predictivity of the model, respectively. A two-tailed heteroscedastic *t-test* with Benjamini-Hochberg correction was also used to determine the statistical significance of differences between the groups. Statistical Total Correlation Spectroscopy (STOCSY), the Human Metabolome Database (HMDB version 3.6, Edmonton, AB, Canada) [26,27] and in-house chemical shift databases were used for metabolite identification.

### 2.7. Gene Expression Analysis

Total RNAs were extracted from cortex and medulla layers of kidney using TRIzol^®^ reagent method. Tissues weighing about 50–100 mg were preserved in TRIzol^®^ and incubated at room temperature (RT) for 3 min, 200 µL of chloroform was added the mixture was shaken vigorously and incubated at RT for 3 min. The reaction was then centrifuged at 12,000× *g* for 15 min at 4 °C. The upper aqueous phase (60%) was transferred to a new 1.5 mL sterile tube, 500 μL of isopropanol was added, mixed well, and then incubated at RT for 10 min and centrifuged at 12,000× *g* for 10 min at 4 °C. The supernatant was discarded while the pellet was washed by 70% ethanol and centrifuged at 7500× *g* for 5 min at 4 °C (twice). The pellet was air dried at RT, re-suspended in 30–50 μL of DEPC water and then the pellet was dissolved by keeping it at 55 °C for 15 min. Total RNA were stored at −80 °C until used.

RNA concentration was measured with OD at 260 nm and reverse-transcribed to complementary DNA (cDNA) using the high capacity reverse transcription Kit (Applied Biosystem, Foster, CA, USA). Obtained cDNA was diluted to 20 ng/μL and stored at −20 °C until used. Gene expression analysis was determined by real-time PCR, using beta-actin (ACTB) as the internal control for normalization [28], in LightCycler^®^ 480 real-time PCR system (Roche Applied Science, Mannheim, Germany). Each PCR condition containing 2.5 μM of primers (forward and reverse primers), cDNA 50 ng/μL, and 2X LightCycler^®^ 480 SYBR green I master mix. The amplification was initiated by pre-incubation at 95 °C for 10 min, followed by 40 cycles at 94 °C for 30 s, 60 °C for 30 s, 72 °C for 1 min, and 79 °C for 0.1 s. Each sample was prepared in duplicate sample and the crossing point (Cp) cycle was calculated and presented as mean ± SEM values. The gene expression levels were determined and the 2^−∆cp^ value; Δcp = Cp_target_ − Cp_actin_ was calculated. Primers for all genes were designed using NCBI with a least one exon-exon junction in the target (Table 1).

### 2.8. Statistical Analysis

The statistical analysis of the urinary analysis and gene expression were reported as mean ± SEM per group of animals and the differences between groups was compared for statistical significance by Student’s t-test. Moreover, all comparisons of gene expression with *p*-values < 0.05 were considered as statistically significant.

## 3. Results

### 3.1. MSG Exhibits Urine Chemistries Similar to Alkaline Loading

**Figure 2:**
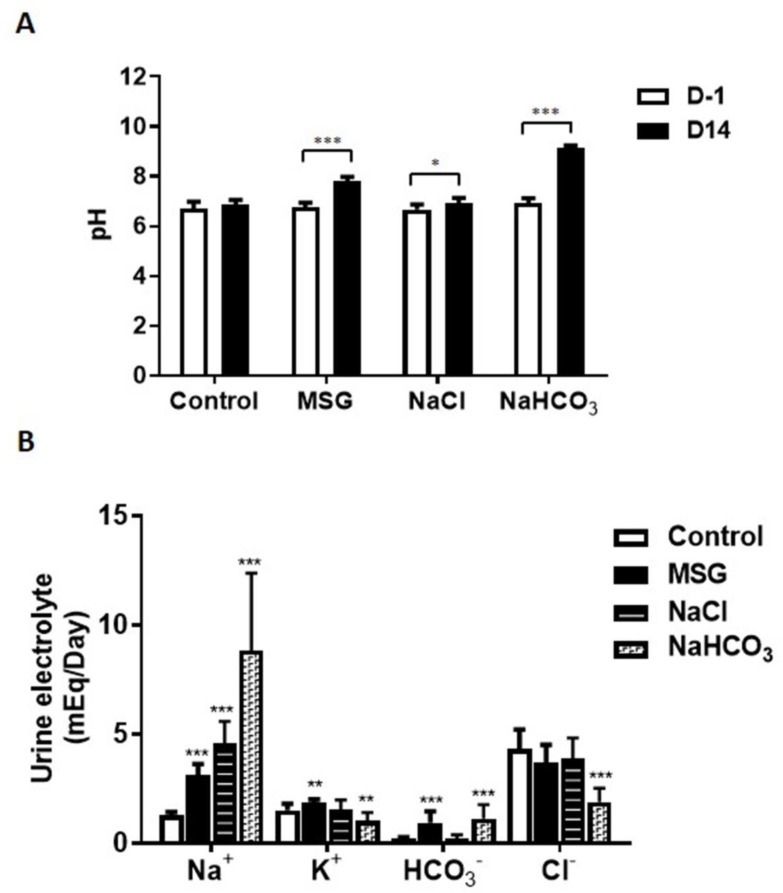
Urine pH (**A**) and electrolytes (**B**) after treatment in male Wistar rats supplemented with 1 g% MSG, 0.34 g% NaCl and 2.4 g% NaHCO_3_ (*n* = 10 per group). Data are shown as mean ± SEM and *p*-values calculated by Student’s *t-test* (* *p* < 0.05, ** *p* < 0.01, *** *p* < 0.001).

After two weeks, urine analyses indicated that the MSG and NaHCO_3_ treatment groups had significantly higher urine pH compared to controls (pH = 7.82, pH = 9.13, and pH = 6.86, respectively; *** *p* < 0.001 for both groups vs. controls), whereas no significant difference in urinary pH was observed between NaCl-treated rats (pH = 6.93) and controls (Figure 2A). Urinary Na^+^ was significantly higher in MSG, NaCl, and NaHCO_3_-treated rats compared to controls (3.1 ± 0.17, 4.59 ± 0.31, 8.84 ± 1.12, 1.28 ± 0.05 mEq/day, respectively). Urinary K^+^ excretion in MSG group (1.83 ± 0.06 mEq/day) was significantly higher, whereas the NaHCO_3_-treated group (1.04 ± 0.12 mEq/day) was the opposite to controls (1.50 ± 0.10 mEq/day). Significantly higher levels of HCO_3_^−^ were also revealed in MSG (0.95 ± 0.16 mEq/day) and NaHCO_3-_treated rats (1.14 ± 0.20 mEq/day) but not in NaCl-treated rats (0.20 ± 0.06 mEq/day) compared to controls (0.19 ± 0.02 mEq/day). No significant differences in urinary Cl^-^ were observed between MSG (3.70 ± 0.26 mEq/day) and NaCl-treated rats (3.85 ± 0.31 mEq/day), whereas significantly lower levels were found in NaHCO_3_-treated rats (1.88 ± 0.20 mEq/day) compared to control rats (4.32 ± 0.28 mEq/day) (Figure 2B).

### 3.2. MSG Suppresses HCO_3_^−^ Reabsorption Similar to Alkaline Loading

**Figure 3:**
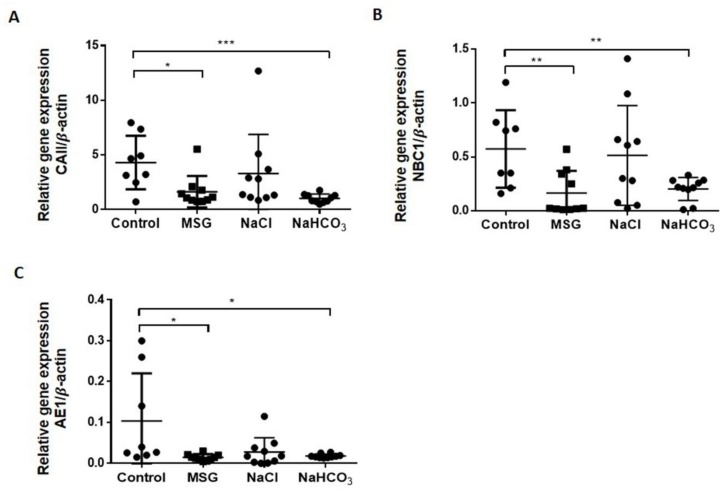
Changes in mRNA expression of ion exchanger genes in cortex layers of rat kidney after 14 days of MSG (*n* = 10), NaCl (*n* = 10) and NaHCO_3_ (*n* = 10) supplementation compared to controls (*n* = 8) (**A**) *CAII*, (**B**) *NBC1*, (**C**) *AE1*. Data are shown as mean ± SEM relative gene expression with beta-actin, * *p* < 0.05; ** *p* < 0.01; *** *p* < 0.001. Abbreviations: *CAII*: carbonic anhydrase2, *NBC1*: Na^+^-HCO_3_^−^ co-transporter1; *AE1*: anion exchanger1.

We used RT-PCR to analyze the expression of the 11 ion-exchanger genes that contribute to the acid-base regulation in the kidney cortex, including the expression of genes related to glutamine/glutamate metabolism and the TCA cycle (Table 1). Three important ion-exchanger genes involved in HCO_3_^−^ reabsorption (*CAII, NBC1* and *AE1*) were suppressed in MSG-treated rats (1.62 ± 0.456, 0.16 ± 0.065, 0.01 ± 0.002, respectively), similar to that of the NaHCO_3_ group (1.02 ± 0.125, 0.20 ± 0.034, 0.02 ± 0.001, respectively) when compared to controls (4.30 ± 0.865, 0.57 ± 0.127, 0.10 ± 0.041, respectively) (Figure 3). No significant differences were observed for *CAII, NBC1,* and *AE1* mRNA expression levels in NaCl-treated rats compared to controls. Glutamine/glutamate metabolism and TCA cycle gene expression in kidney were unchanged in MSG, NaCl and NaHCO_3_ compared to control groups (data shown in Supplementary Materials).

### 3.3. MSG Shares Common Urinary Metabolic Profile Resembling Alkaline Loading

**Figure 4:**
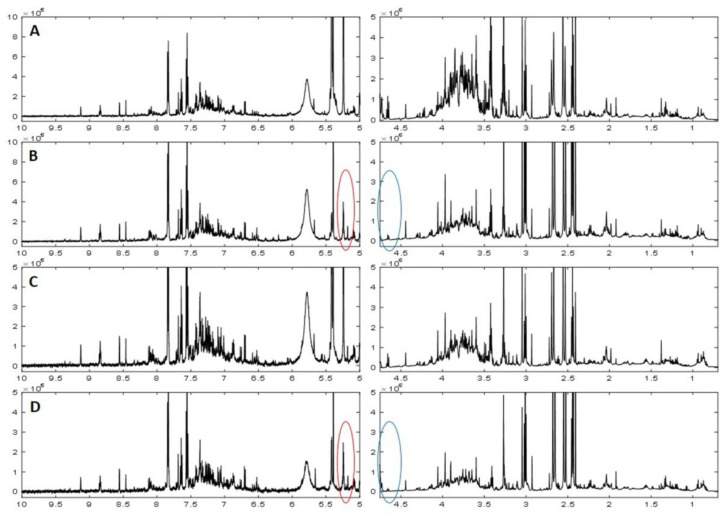
The 600 MHz ^1^H-NMR spectra of urinary samples collected over 24 h at day 14 from (**A**) control, (**B**) MSG-fed, (**C**) NaCl-fed, and (**D**) NaHCO_3_ –fed rats (*n* = 10 per group).

**Figure 5:**
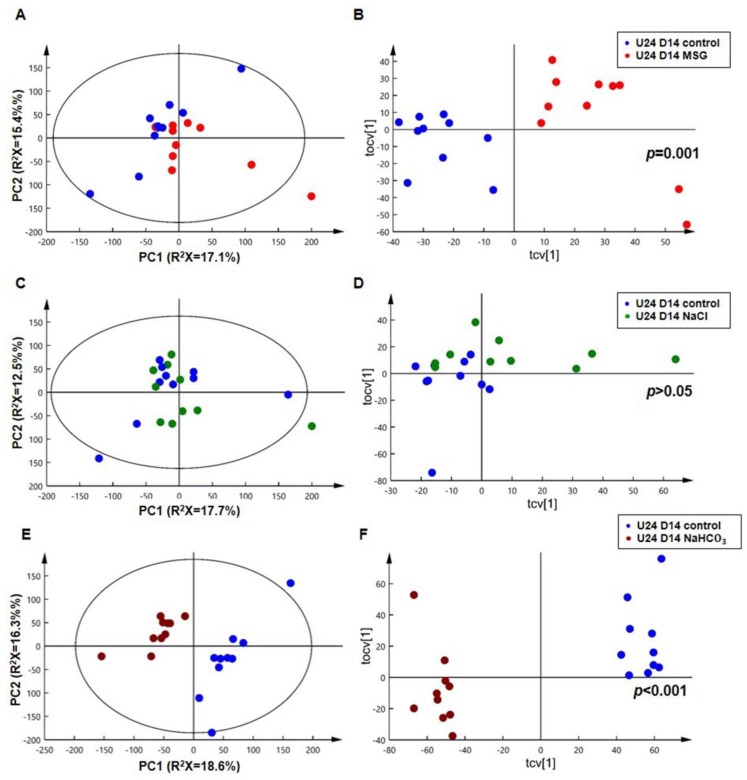
PCA scores plots (left panel) and O-PLS-DA cross-validated scores plots (right panel) of 24-h urine at day 14 (U24 D14). (**A**,**B**): control (blue) vs. MSG (red); (**C**,**D**): control (blue) vs. NaCl (green); (**E**,**F**): control (blue) vs. NaHCO_3_ (brown) (*n* = 10 per group).

^1^H-NMR spectra of 24 h urine samples from control, MSG, NaCl and NaHCO_3_-treatment groups at day 14 (D14) are plotted in Figure 4. Differences in raw spectra between 4.5 ppm and 5.25 ppm were observed in MSG and NaHCO_3_-treated rats compared to NaCl and control rats (Figure 4). Urinary NMR spectral data was analyzed using PCA and the scores plot is shown in Figure 5. No clustering based on the treatment group was observed in the PCA scores plot (Figure 5A). However, the O-PLS-DA cross-validated scores plot shows a clear separation between controls and MSG at D14 (Figure 5B) with a permutation p-value of 0.001, R^2^X of 44%, and Q^2^Y of 0.75. Based on PCA and O-PLS-DA, no clustering or separation was observed between controls and the NaCl-treated group (Figure 5C,D). In contrast, clear clustering and complete separation were observed based on PCA and O-PLS-DA, between controls and the NaHCO_3_-treated group (Figure 5E,F) with a permutation p value of 0.001, R^2^X of 47%, and Q^2^Y of 0.95.

The detected urinary metabolite changes are summarized in Table 2 and changes in the urinary profiles were observed in MSG and NaHCO_3_-treated rats when compared with control animals. In particular, nine urinary metabolites, including glutamate, citrate, malonate, alpha-ketoglutarate, beta-hydroxyisovalerate, 5-aminovalerate, 5-hydroxymethyl-4-methyluracil, dimethylamine, and methylamine were significantly higher in the MSG treatment group, whereas taurine was significantly higher in control animals. Seven urinary metabolites, including glutamate, citrate, malonate, alpha-ketoglutarate, 5-aminovalerate, beta-hydroxyisovalerate, and taurine found in the MSG group were also observed in theNaHCO_3_ group. However, three metabolites, i.e., 3-carboxy-2-methyl-3-oxopropanamine, succinate, and choline were significantly higher in the NaHCO_3_ group compared to controls. The 5-hydroxymethyl-4-methyluracil metabolite was significantly altered in both MSG and NaHCO_3_, but in opposite ways. The similar effects of MSG and NaHCO_3_ supplementation on urine pH, urine electrolytes, ion exchanger gene expression and urinary metabolic markers are illustrated in Table 3.

## 4. Discussion

The kidney plays a dominant role in maintaining the homeostasis of plasma and urine pH by acid excretion and HCO_3_^−^ reabsorption via ion exchangers. We report for the first time that short-term MSG consumption can induce alkaline urine similar to the NaHCO_3_ supplementation group, and the MSG group has a similar metabolic profile with potentially similar clinical consequences.

First, we believe that the increased urinary pH in the MSG group is secondary to its glutamate composition, not to sodium itself. This is supported by the urinary pH of the NaCl group which does not increase as the amount of Na intake increased compared to the MSG group (Figure 2B). Our investigation of MSG-induced alkaline urine confirms previous data from rats receiving 20% dietary MSG for 5 weeks [29], fed 6% MSG for 3 months [30], or receiving 2 mg/g body weight MSG/day in drinking water for 9 months [14]. We also demonstrated that the MSG-treated group has similar urine electrolyte levels when compared to the NaHCO_3-_treated group. MSG-treated rats have significantly higher urinary Na^+^, K^+^ and HCO_3_^−^, as observed with longer supplementation [14]. The excretion of Na^+^, K^+^ and HCO_3_^−^ in MSG-treated rats may suggest these elements are excessively uptaken or overproduced by metabolic pathways. Our current hypothesis is that the excess of Na^+^ derives directly from the overconsumption of MSG as show in the NaCl group, whereas HCO_3_^−^ and K^+^ derive from the catabolism of glutamate and other nutrient metabolism. The byproduct of glutamate metabolism might be similar to that of potassium citrate that generates HCO_3_^−^ via its catabolic citric acid cycle, leading to the alkaline urinary pH [31]. Alkaline urine may influence the kidney’s capacity to secrete or reabsorb metabolites leading to the suppression of bicarbonate reabsorption in the kidney.

Second, the expression of ion exchangers involved in bicarbonate reabsorption in the kidney cortex of MSG-treated rats is decreased (Figure 3) and this indicates lower bicarbonate reabsorption in MSG-treated rats, similar to that of the NaHCO_3_ supplemented group, which was not observed in NaCl-treated animals. The decreased NBC1 expression in the proximal tubule has been reported in rats treated with NaHCO_3_ loading [32] while the immunostaining intensity of AE1 was increased in metabolic acidosis and reduced in metabolic alkalosis [33].

Third, the ^1^H-NMR-based metabolomics approach revealed that the MSG-induced urinary metabolic profiles were similar to the NaHCO_3_ group. In particular, MSG consumption generates a pattern of metabolites with higher levels of glutamate, alpha-ketoglutarate, malonate, citrate, beta-hydroxyisovalerate and 5-aminovalerate, whereas the level of taurine is lower than rats receiving normal drinking water, as also observed in rats receiving NaHCO_3_. A quick observation of the four treatment groups showed differences in the raw spectra between 4.5 ppm and 5.25 ppm in MSG and NaHCO_3-_treated rats compared to NaCl and control rats (Figure 4). In agreement with these raw spectra, data analysis using PCA and O-PLS-DA revealed that both MSG and NaHCO_3_ treatments show a clustering difference between treatment and control rats (Figure 5).

Ten metabolites that relate to MSG consumption are linked to catabolism of amino acids, fatty acids, vitamin, pyrimidine and citric acid cycle. For example, alpha-ketoglutarate and citrate are intermediates of the citric acid cycle. Alpha-ketoglutarate and glutamate are from transamination reactions. Beta-hydroxyisovalerate and 5-aminovalerate are leucine and lysine degradation products, respectively. Malonate is from either aspartate or fatty acid catabolism. Taurine is from cysteine or serine metabolism whereas dimethylamine and methylamine are from choline metabolism. Lastly, 5-hydroxymethyl-4-methyluracil, a marker of DNA damage, is from pyrimidine catabolism [34].

The characterization of the 10 metabolites observed in the MSG treatment group and the 11 metabolites in the NaHCO_3_ treatment group, with some shared changes (Table 2), warrants a more detailed discussion. The higher levels of amino acid (glutamate) and amino acids-related metabolites (alpha-ketoglutarate, malonate, citrate, beta-hydroxyisovalerate and 5-aminovalerate) found in the urine of MSG and NaHCO_3_-treated groups compared to controls may be related to their urine alkalinizing condition. Glutamate kidney re-uptake requires glutamate transporters [35], which co-transport H^+^ with glutamate, and high urinary pH may inhibit glutamate re-uptake in the renal brush border, thus contributing to the higher urinary glutamate. Alkaline urine also inhibits metabolite reabsorption in the case of alpha-ketoglutarate, malonate and citrate in the MSG and NaHCO_3_-treated rats. Based on their pKa, the alkaline urine may deprotonate alpha-ketoglutarate^1-^, malonate^1−^, and citrate^2−^ to form alpha-ketoglutarate^2−^, malonate^2−^, and citrate^3−^, respectively, and affect their reabsorption [36,37]. In metabolic/respiratory alkalosis, alpha-ketoglutarate excretion increases to levels that are several times above normal [38,39,40]. Under alkaline loading conditions, the blood concentration of alpha-ketoglutarate rises and net alpha-ketoglutarate reabsorption in the proximal tubule and Henle’s loop is decreased, making the alpha-ketoglutarate secretion increase in the same nephron segments, and leading to a significant increase in the urinary excretion of alpha-ketoglutarate [41,42]. Moreover, citrate, alpha-ketoglutarate, and succinate competitively inhibit the transport of each other [43].

A similar scenario is also hypothesized for malonate as citrate and malonate compete for reabsorption in the renal tubular cell [44]. The higher level of a lysine degradation product, 5-aminovalerate and leucine degradation product, beta-hydroxyisovalerate found in MSG and NaHCO_3_ supplemented animals may relate to tissue injury. The beta-hydroxyisovalerate itself is a metabotoxin [45]. Taurine is a normal constituent of human urine, however, its level varies markedly among individuals depending on age, hormones, stress, and diet [46], however, our control and MSG, NaHCO_3_ groups had comparable characteristics, except for MSG and NaHCO_3_ intake. The decrease in taurine in the MSG and NaHCO_3_ groups may relate to either taurine use, secondary to oxidative stress, or to its degradation, as observed in chronic renal failure [47], glomerulonephritis, diabetic nephropathy, chronic renal failure, and acute kidney injury [48].

In fact, excessive renal metabolism of glutamate can be a source of ROS. Moreover, chronic MSG intake is associated with decreased levels of antioxidant enzymes and increased lipid peroxidation; leading to cellular and functional damage of the kidneys [49]. Such effects are not observed with sodium bicarbonate (NaHCO_3_). The distinct urinary metabolites between MSG and NaHCO_3_ are methylamine and dimethylamine, which were only observed in MSG-treated animals. Methylamine and dimethylamine are metabolites from the gut microbiota [50,51]. This gut microbiota is also found in human intestine [52]. MSG may alter the gut microbiota community, specifically those that are involved in methylamine metabolism. However, the connection of MSG consumption and methylamine metabolism needs to be further explored.

In summary, MSG consumption may resemble alkali loading (Table 3), causing suppression of ion exchangers responsible for bicarbonate absorption which correspond to the higher excretion of urinary bicarbonate in MSG and NaHCO_3_-treated rats. MSG consumption exhibits similar features to alkaline loading, causing alkaline urine, leading to alteration of di- and tricarboxylic metabolites, i.e., glutamate, alpha-ketoglutarate, citrate, and malonate reabsorption in the kidney. We may also speculate that MSG itself may alter the gut microbiome to catabolize dietary molecules, such as amino acids and choline and urinary metabolites could be potential markers for MSG consumption in animals and should now be studied in humans. However, there are two limitations in this present study to be aware of: the effects of MSG consumption were only tested in male animals, and the frequency of obtaining MSG was every time the animals drunk water. This behavior differs from humans because humans receiving MSG only consume MSG at their regular meal times, which is usually 2-3 times a day.

## 5. Conclusions

Short-term MSG consumption exhibits similar effects as consuming NaHCO_3_, an alkalinizing agent; it induces alkaline urine and alters the ion-exchanger gene expression that relates to bicarbonate kidney reabsorption. This implies that MSG consumption induces metabolic changes with a pattern of urinary metabolites, which may be used for monitoring MSG exposure in humans.

## Figures and Tables

**Table 1:** Oligonucleotide primers used for gene expression analysis.

Group	Gene	Primer	Oligonucleotide Sequence	Product Size (bp)
Internal Control	*Beta-actin*	Forward	ACAACCTTCTTGCAGCTCCT	197
		Reverse	ACCCATACCCACCATCACAC	
Ion-Exchanger	*AE1*	Forward	TCCCGCTACACTCAGGAGAT	118
		Reverse	CAGGGGCATAGCTCTCTTGT	
	*CAII*	Forward	TGCTGGAATGTGTGACCTGG	101
		Reverse	CTCCCCCTCCGAATTGAAGT	
	*Na^+^/K^+^ ATPase*	Forward	CAGCACTCGCTTTCCCTCG	189
		Reverse	GGCCAGGCAGCCATAGAATA	
	*H^+^/K^+^ ATPase*	Forward	CCCCTGAGTACGTGAAGTTCG	168
		Reverse	CCACAACCACAGCAATGAGTG	
	*CA IV*	Forward	GTCTATGCCCTCAAGCACCA	114
		Reverse	TTGGGCTCCTTGGCTTGAAT	
	*NBC1*	Forward	GCTATCCCGGCTTTGCTAGT	153
		Reverse	GAAGGAGCACACCACCATGA	
	*NHE3*	Forward	ACTGCTTAATGACGCGGTGA	160
		Reverse	GAAGGCGAAGATGACACCA	
	*Rhbg*	Forward	TGTCCGCTACAACCACGAAA	96
		Reverse	TGGAAGCTTGGGTAGCGAAA	
	*Rhcg*	Forward	CTCTTCGGCGTGTTCGTGC	200
		Reverse	CCTACAGCGCTGAACCCATA	
	*Pendrin*	Forward	AGAACCAGGCCAAATCCAGG	83
		Reverse	CAAGTCTACGCATGGCCTCA	
	*H^+^-ATPase*	Forward	TGCCTTCAGTTAGAGAGGCCGTGA	147
		Reverse	TGCCAAGAAGAGTCTGGGACAAGG	
Glutamate and Glutamine Metabolism	*XC-sys*	Forward	GCATCGTCCTTTCAAGGTGC	150
		Reverse	AAGAGGTAATACGCCGGGAC	
	*EAAC1*	Forward	AAACCACGGTGCTCGGTC	127
		Reverse	ACCGGCGTTTGTGAGGAATC	
	*Glutaminase*	Forward	TGGGCATGATGTGTTGGTCT	199
		Reverse	TACGCAGCAAACAGGAGGTT	
	*SNAT3*	Forward	GGAACGGAGTGCTGAACGTG	83
		Reverse	CTGAAACCACCCCAGAGCAC	
	*PEPCK*	Forward	TGCCATGGCTGCTATGTACC	89
		Reverse	TTTGGATGCTACGGCATGGT	
TCA Cycle	*Citrate synthase*	Forward	TGCTACACAGAACCTCAGTTCAC	243
		Reverse	ATCTGACACGTCTTTGCCGA	
	*Aconitase*	Forward	CCTGTACCTGACACTGCTCG	223
		Reverse	TGTAGTCAGAGGGGTCAGCA	
	*IDH*	Forward	TGCAAAAATATCCCCCGCCT	144
		Reverse	GCCATCCTTTGGGGTGAAGA	

Abbreviations: *AE1*: anion exchanger1, *CAII*: carbonic anhydrase2, *CAIV*: carbonic anhydrase4, *NBC1*: Na^+^-HCO_3_^−^ co-transporter1, *NHE3*: Na^+^/H^+^ exchanger3, *Rhbg:* Rh family B glycoprotein, *Rhcg*: Rh family C glycoprotein, *XC-sys*: Cysteine/glutamate transporter, *EAAC1*: Excitatory amino acid transporter1, *SNAT3*: Na^+^-coupled neutral amino acid transporter3, *PEPCK*: Phosphoenolpyruvate carboxykinase, *IDH*: Isocitrate dehydrogenase.

**Table 2:** Relative changes at 2 weeks of 24 h urine metabolites in control, MSG and NaHCO_3_ rats (*n* = 10 per group) using the ^1^H-NMR profiles.

Metabolites	Chemical Shift (Multiplicity)	MSG and NaHCO_3_ Induced Metabolic Changes Compared to Control		The Acid Dissociation Constant (pKa)
		(−) Control vs. (+) MSG R2X = 44%, Q2Y = 0.75, *p* = 0.001	(−) Control vs. (+) NaHCO_3_ R2X = 47%, Q2Y = 0.95, *p* = 0.001	
3-carboxy-2-methyl-3-oxopropanamine	**1.08 (d);** 2.49 (m); 3.19(m); 3.56 (m); 3.72 (m)	-	(*0.95)(∆3.33 × 10^−10^)	
Beta-hydroxyisovalerate	1.28 (s)	(*0.77)(∆6.19 × 10^−5^)	(*0.89)(∆1.83 × 10^−7^)	pKa_1_ = 4.55
5-aminovalerate	1.68 (m); **2.21 (t);** 3.02 (t)	(*0.79)(∆3.35 × 10^−5^)	(*0.95)(∆1.38 × 10^−10^)	pKa_1_ = 4.27, pKa_2_ = 10.77
5-hydroxymethyl-4-methyluracil	**1.98 (s);** 4.42 (s)	(*0.83)(∆5.84 × 10^−6^)	(*−0.84)(∆4.15 × 10^−6^)	pKa_1_ = 9.87
Glutamate	**2.02 (m);** 2.34 (m); 3.76 (m)	(*0.85)(∆2.31 × 10^−6^)	(*0.88)(∆2.91 × 10^−7^)	pKa_1_ = 2.19, pKa_2_ = 4.25, pKa_3_ = 9.67
Succinate	2.41 (s)	-	(*0.87)(∆8.10 × 10^−7^)	pKa_1_ = 4.16, pKa_2_ = 5.61
Alpha-ketoglutarate	**2.44 (t);** 3.01 (t)	(*0.75)(∆1.37 × 10^−4^)	(*0.89)(∆1.46 × 10^−7^)	pKa_1_ = 2.47, pKa_2_ = 4.68
Citrate	**2.54 (d);** 2.66 (d)	(*0.87)(∆5.10 × 10^−7^)	(*0.96)(∆4.63 × 10^−11^)	pKa_1_ = 3.14, pKa_2_ = 4.77, pKa_3_ = 6.39
Methylamine	2.61 (s)	(*0.52) (∆0.019)	-	pKa_1_ = 10.63
Dimethylamine	2.77 (s)	(*0.71)(∆4.76 × 10^−4^)	-	pKa_1_ = 2.36, pKa_2_ = 10.21
Malonate	3.11 (s)	(*0.74)(∆2.17 × 10^−4^)	(*0.97)(∆1.66 × 10^−12^)	pKa_1_ = 2.85, pKa_2_ = 5.70
Choline	**3.21 (s);** 3.52 (m); 4.07 (m)	-	(*0.80)(∆2.70 × 10^−5^)	pKa_1_ = 13.9
Taurine	**3.25 (t);** 3.43 (t)	(*−0.73)(∆2.69 × 10^−4^)	(*−0.80)(∆2.80 × 10^−5^)	pKa_1_ = 1.15, pKa_2_ = 9.06

R2X and Q2Y show the variance explained and predicted by each model while *P* values for all models were derived from the permutation test (*n* = 1000). (+) indicates a higher correlation, whereas (−) indicates a lower correlation of urinary metabolite after MSG and NaHCO_3_ consumption. (*) represents the correlation value and (∆) represents the *p* value of the specific peak. The bolded chemical shift per metabolite was used as the STOCSY driver peak and for deriving the correlation and *p*-value. (* *p* < 0.05, ** *p* < 0.01, *** *p* < 0.001*).* Abbreviations: s, singlet; d, doublet; t, triplet; m, multiplet, pKa; acid dissociation constant. Note; (−) Control vs. (+) NaCl, R2X = 38%, Q2Y = 0.12, *p* = 0.433.

**Table 3:** The similar effects of MSG and NaHCO_3_ supplementation on urine pH, urine electrolytes, ion exchanger gene expression and urinary metabolic markers.

	Control	MSG	NaCl	NaHCO_3_
Urine pH	-	↑	-	↑
Urine Electrolytes (HCO_3_^−^)	-	↑	-	↑
Ion exchanger gene expression (HCO_3_^−^ reabsorption, *CAII, NBC1, AE1*)	-	↓	-	↓
Urinary metabolic markers Beta-hydroxyisovalerate 5-aminovalerate Glutamate Alpha-ketoglutarate Citrate Malonate	-	↑	-	↑
Taurine	-	↓	-	↓

## Supplementary Materials

The following are available online at https://www.mdpi.com/2218-273X/9/10/542/s1, Figure S1–S3. Changes in mRNA expression of ion exchanger genes in the cortex (left panel) and medulla (right panel) layers of rat kidney after 14 days of MSG, NaCl and NaHCO_3_ supplementation compared to controls; Figure S4. Changes in mRNA expression of glutamate and glutamine metabolism in the cortex (left panel) and medulla (right panel) layers of rat kidney after 14 days of MSG, NaCl and NaHCO_3_ supplementation compared to control; Figure S5. Changes in mRNA expression of TCA cycle in the cortex (left panel) and medulla (right panel) layers of rat kidney after 14 days of MSG, NaCl and NaHCO_3_ supplementation compared to controls.
