# Supplementary material for: Monosodium Glutamate (MSG) Renders Alkalinizing Properties and Its Urinary Metabolic Markers of MSG Consumption in Rats
Source: Biomolecules. 2019 Sep 27;9(10):542. doi: 10.3390/biom9100542 (PMC6843139; doi:10.3390/biom9100542)
Supplement: Supplementary file 1 [file biomolecules-09-00542-s001.pdf]

Article

Monosodium glutamate (MSG) renders alkalinizing properties and its urinary metabolic markers of MSG consumption in rats

Kanokwan Nahok[1,7], Jia V. Li[2,3], Jutarop Phetcharaburanin[1], Hasina Abdul[2], Chaisiri Wongkham[1], Raynoo Thanan[1], Atit Silsirivanit[1], Sirirat Anutrakulchai[4,7], Carlo Selmi[5,6*] & Ubon Cha'on[1,7*]

1. Department of Biochemistry, Faculty of Medicine, Khon Kaen University, Khon Kaen, 40002, Thailand.
2. Department of Metabolism, Digestive Disease and Reproduction, Faculty of Medicine, Imperial College London, South Kensington, London, SW7 2AZ, United Kingdom.
3. Centre for Digestive and Gut Health, Institute of Global Health Innovation, Imperial College London, SW7 2AZ, United Kingdom.
4. Department of Medicine, Faculty of Medicine, Khon Kaen University, Khon Kaen, 40002, Thailand.
5. Rheumatology and Clinical Immunology, Humanitas Clinical and Research Center, Milan, 20089, Italy
6. BIOMETRA Department, University of Milan, Milan, 20089, Italy.
7. Chronic Kidney Disease prevention in the Northeast Thailand (CKDNET), Khon Kaen University, 40002, Thailand.

* Correspondence: ubocha@kku.ac.th and carlo.selmi@humanitas.it

Supplementary data: mRNA expression genes of ion-exchanger, glutamate/glutamine metabolism and TCA cycle in rat kidney

We used RT-PCR to screen the expression of the 11 ion-exchanger genes that contribute to the acid-base regulation in the kidney cortex, including the expression of 5 genes related to glutamine/glutamate metabolism and 3 genes that involved TCA cycle **(Table 1).** The results reveled that MSG-treated rats had significantly decreased expression of ion-exchanger genes, namely CAII, NBC1, and AE1, involved in the bicarbonate kidney reabsorption similar to $NaHCO_3$. No significant differences were observed for mRNA expression levels of another ion-exchanger genes both cortex and medulla layer **(Figure S1-S3).** Glutamine/glutamate metabolism **(Figure S4)** and TCA cycle **(Figure S5)** gene expression in kidney were unchanged in MSG, NaCl and $NaHCO_3$ compared to control groups.

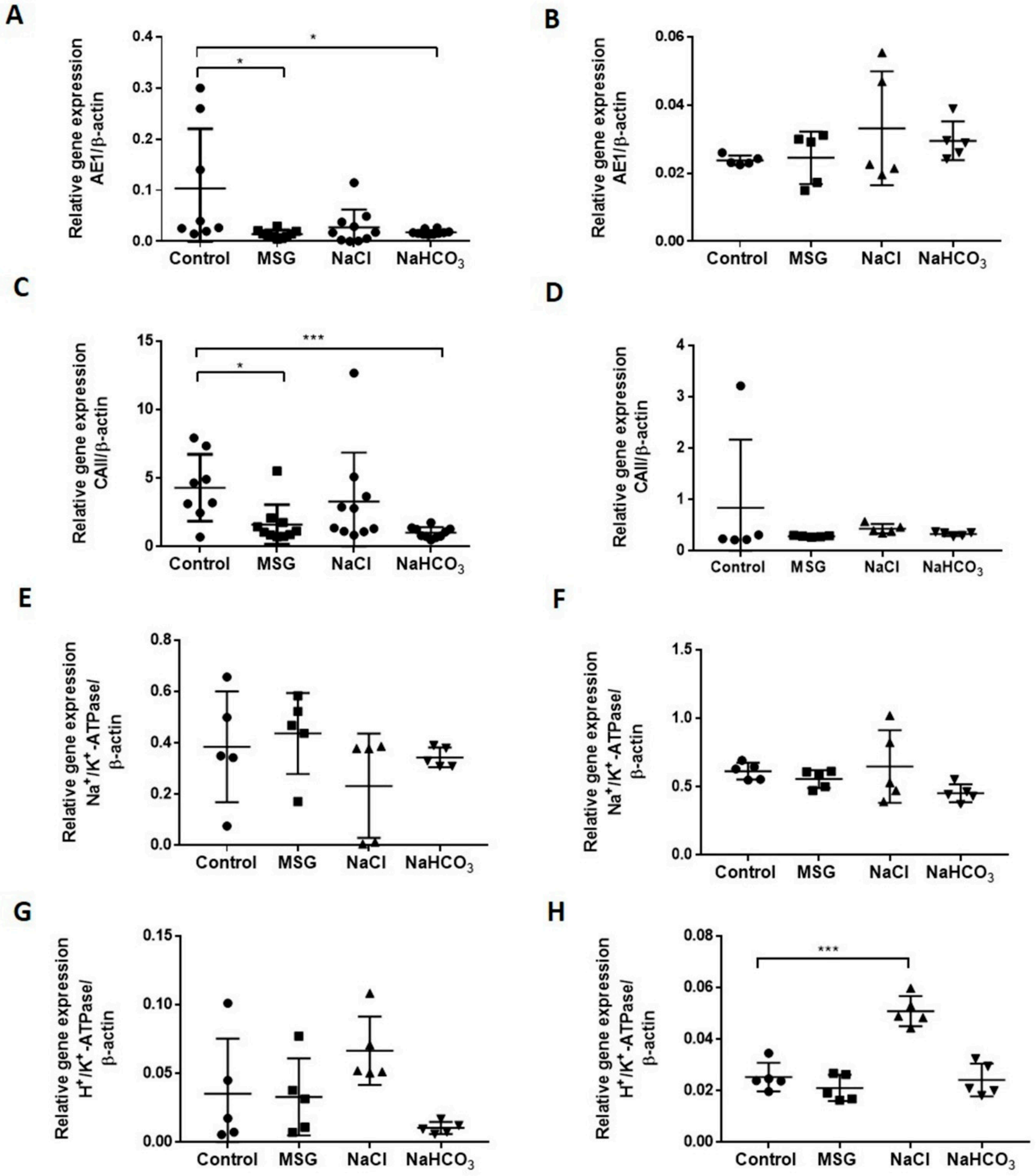

Figure S1. Changes in mRNA expression of ion exchanger genes in the cortex (left panel) and medulla (right panel) layers of rat kidney after 14 days of MSG, NaCl and $NaHCO_3$ supplementation compared to controls. (A-B) AE1, (C-D) CAII, (E-F) Na^+/K^+ -ATPase, (G-H) H^+/K^+-ATPase. Data are shown as mean ± SD relative gene expression with beta-actin, **p*< 0.05; ***p*< 0.01; ****p*< 0.001.

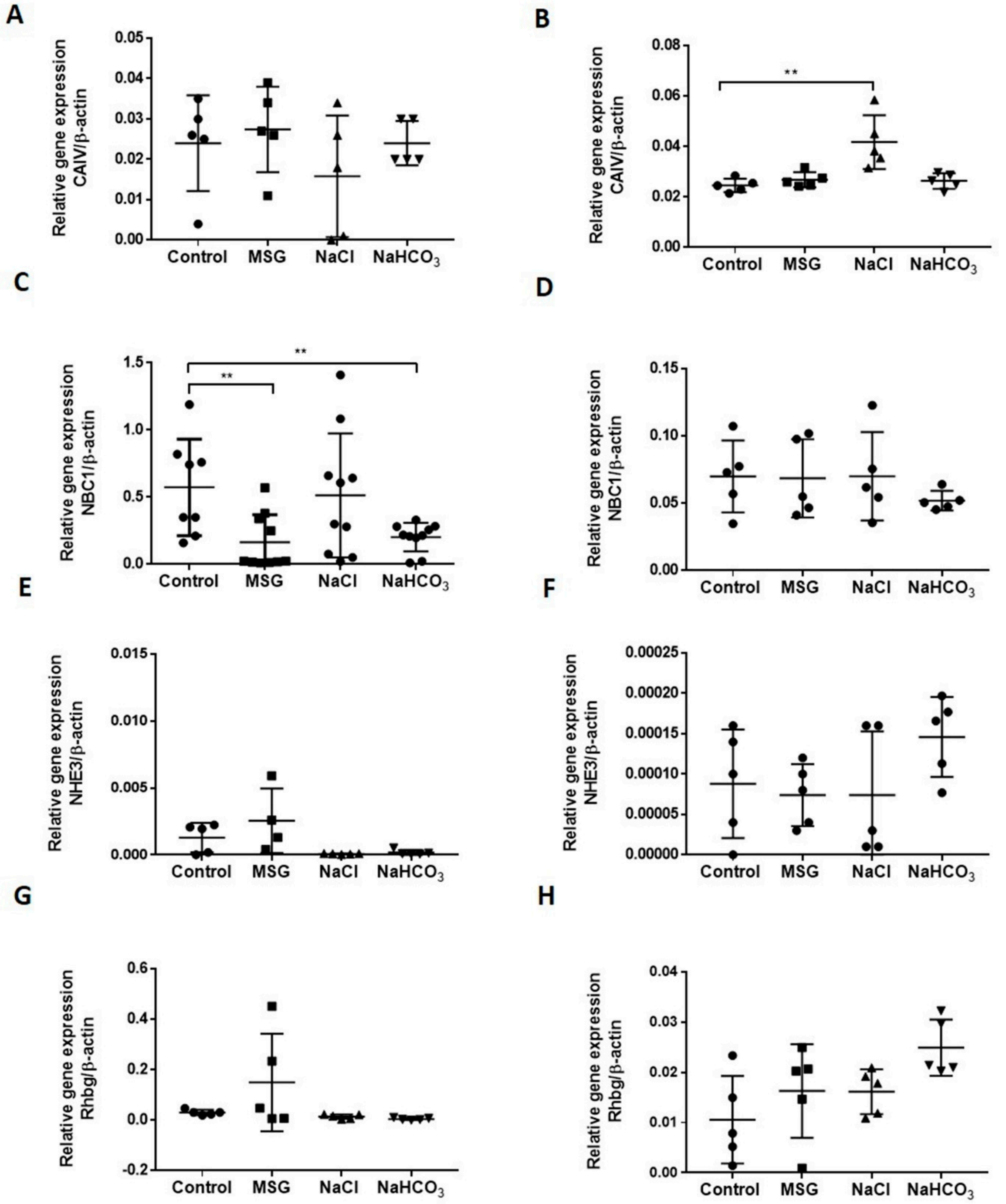

Figure S2. Changes in mRNA expression of ion exchanger genes in the cortex (left panel) and medulla (right panel) layers of rat kidney after 14 days of MSG, NaCl and $NaHCO_3$ supplementation compared to controls. (A-B) CAIV, (C-D) NBC1, (E-F) NHE3, (G-H) Rhbg. Data are shown as mean ± SD relative gene expression with beta-actin, ***p*< 0.01.

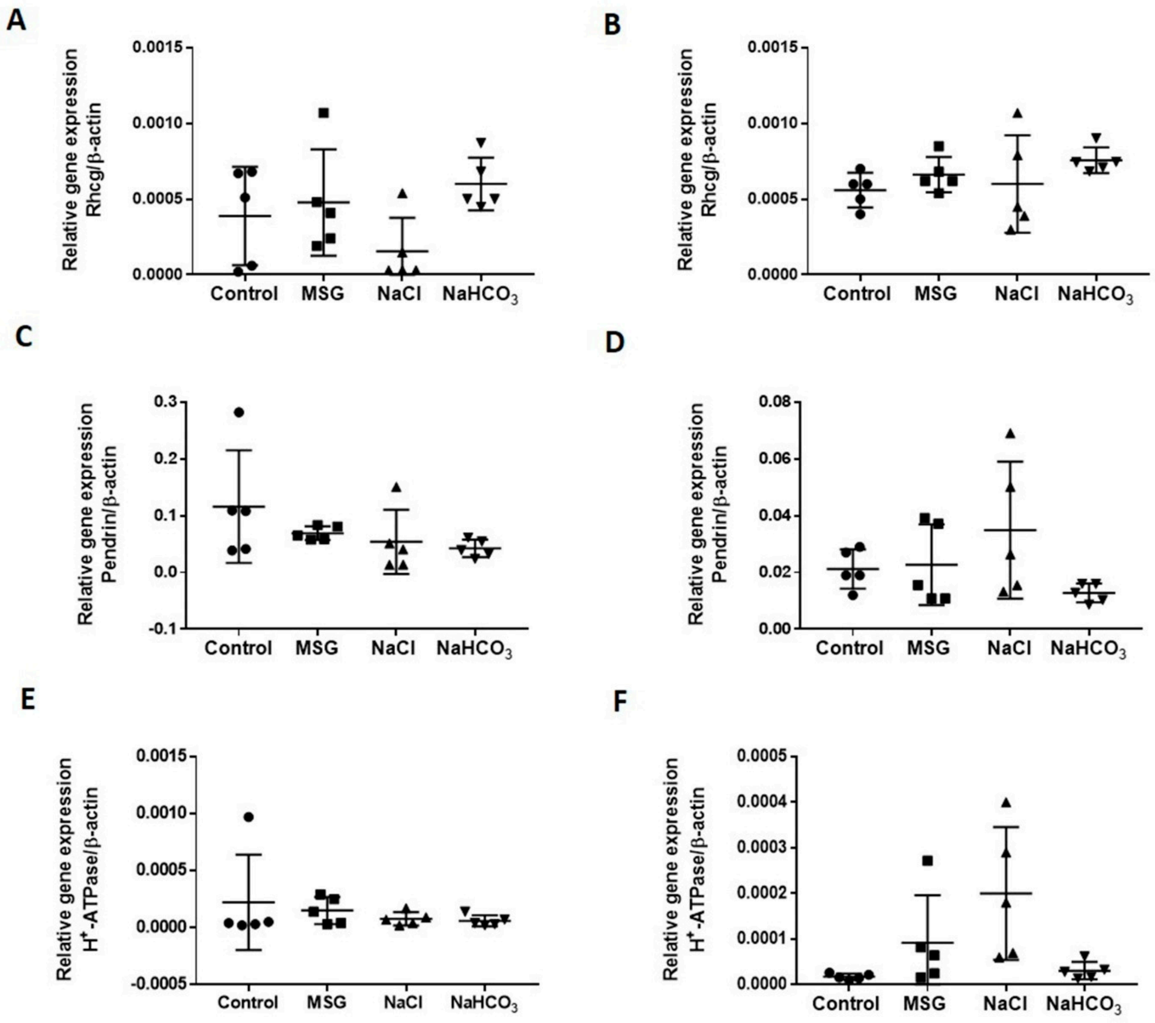

Figure S3. Changes in mRNA expression of ion exchanger genes in the cortex (left panel) and medulla (right panel) layers of rat kidney after 14 days of MSG, NaCl and $NaHCO_3$ supplementation compared to controls. (A-B) Rhcg, (C-D) Pendrin, (E-F) H^+-ATPase. Data are shown as mean ± SD relative gene expression with beta-actin.

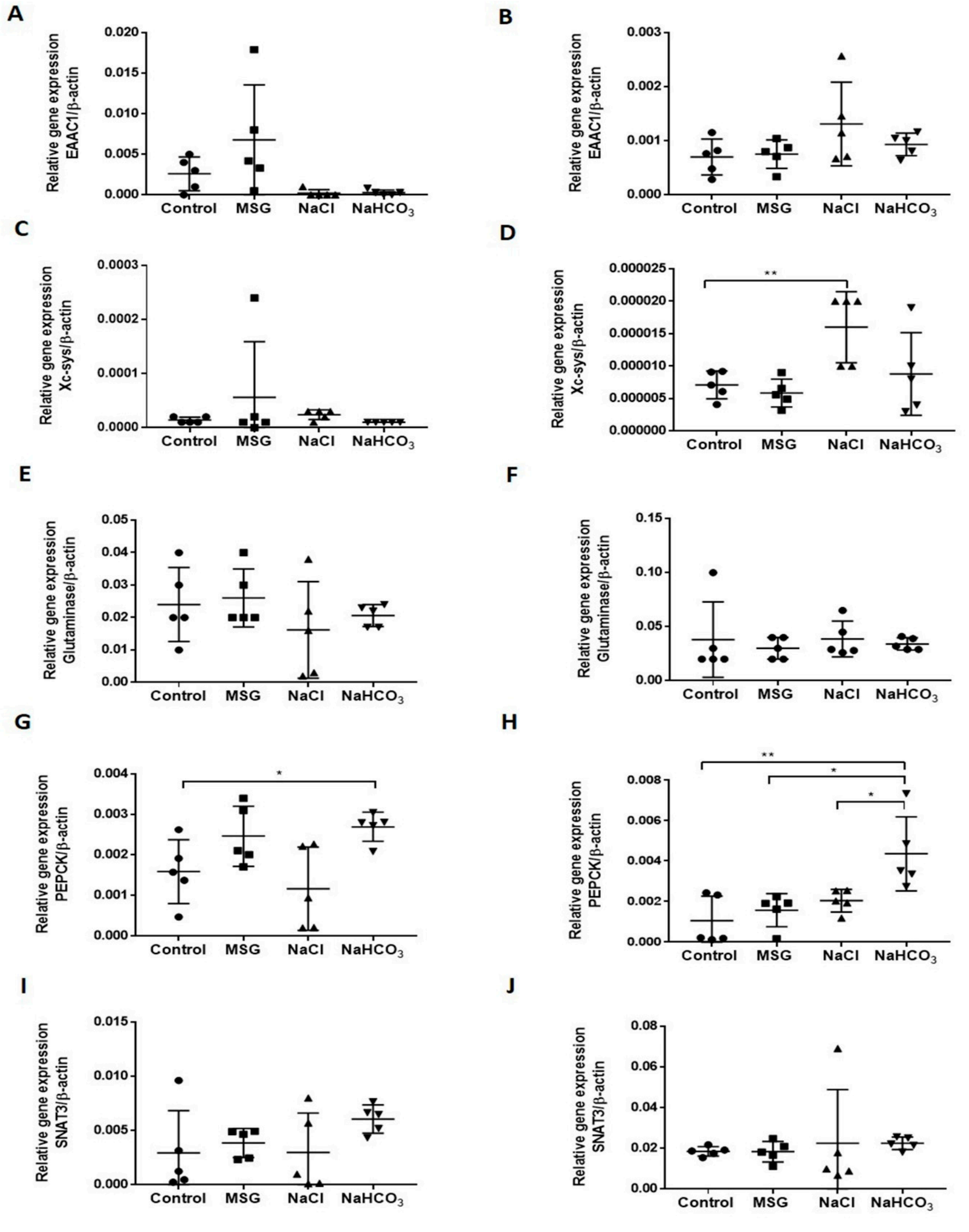

Figure S4. Changes in mRNA expression of glutamate and glutamine metabolism in the cortex (left panel) and medulla (right panel) layers of rat kidney after 14 days of MSG, NaCl and $NaHCO_3$ supplementation compared to controls. (A-B) EAAC1, (C-D) XC-sys, (E-F) Glutamanase, (G-H) PEPCK, (I-J) SNAT3. Data are shown as mean ± SD relative gene expression with beta-actin, *$p < 0.05$; **$p < 0.01$; ***$p < 0.001$.

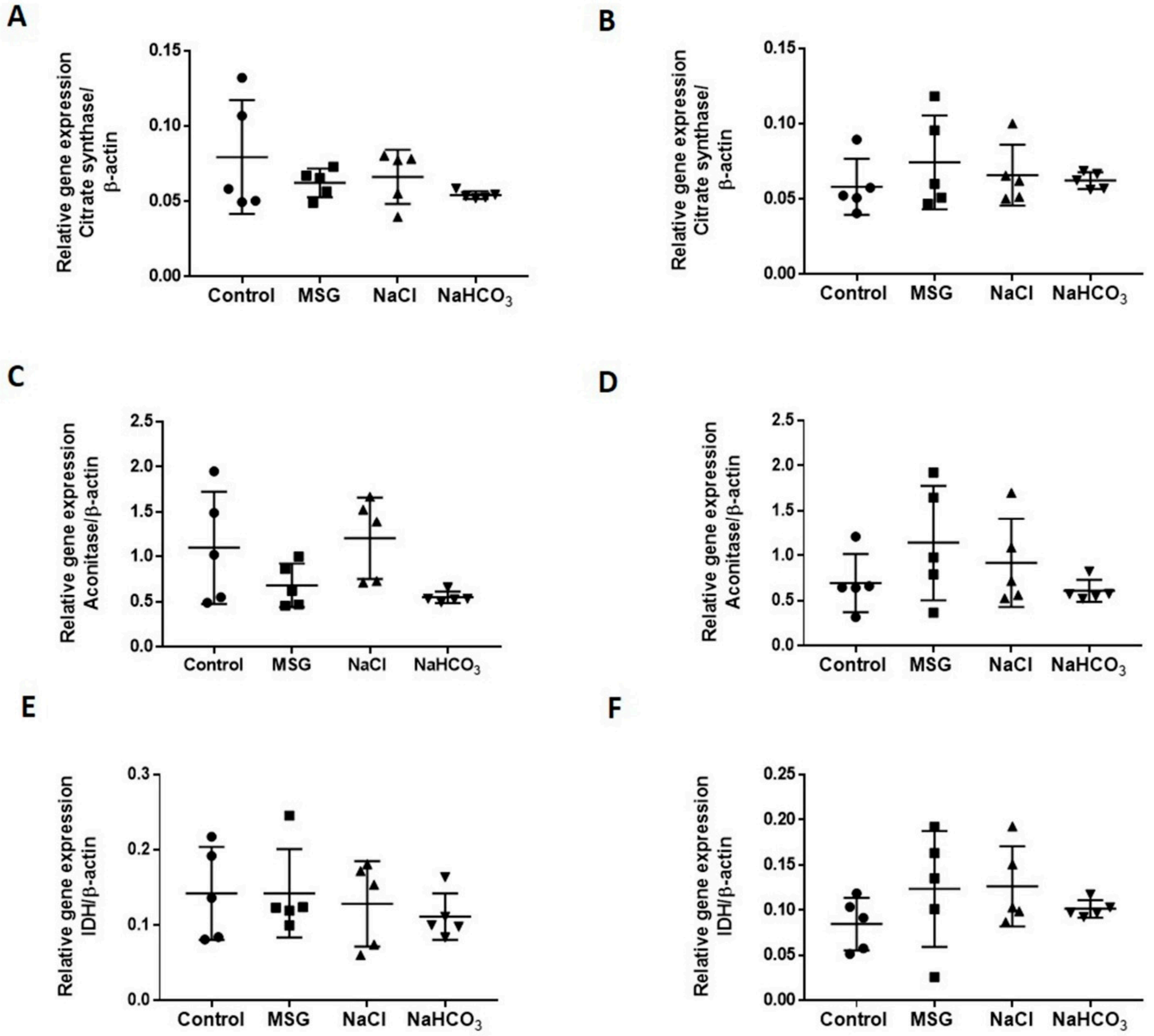

Figure S5. Changes in mRNA expression of TCA cycle in the cortex (left panel) and medulla (right panel) layers of rat kidney after 14 days of MSG, NaCl and $NaHCO_3$ supplementation compared to controls. (A-B) Citrate synthase, (C-D) Aconitase, (E-F) IDH. Data are shown as mean ± SD relative gene expression with beta-actin.
